# Survival-Based Biomarker Module Identification Associated with Oral Squamous Cell Carcinoma (OSCC)

**DOI:** 10.3390/biology10080760

**Published:** 2021-08-08

**Authors:** Prithvi Singh, Arpita Rai, Amit Kumar Verma, Mohammed A. Alsahli, Arshad Husain Rahmani, Saleh A. Almatroodi, Faris Alrumaihi, Kapil Dev, Anuradha Sinha, Shweta Sankhwar, Ravins Dohare

**Affiliations:** 1 Centre for Interdisciplinary Research in Basic Sciences, Jamia Millia Islamia, New Delhi 110025, India; prithvi.mastermind@gmail.com; 2 Department of Oral Medicine and Radiology, Dental Institute, Rajendra Institute of Medical Sciences, Bariatu, Ranchi 834009, India; arai@jmi.ac.in; 3 Department of Biotechnology, Faculty of Natural Sciences, Jamia Millia Islamia, New Delhi 110025, India; averma2@jmi.ac.in; 4 Department of Medical Laboratories, College of Applied Medical Sciences, Qassim University, Buraydah 51452, Saudi Arabia; shly@qu.edu.sa (M.A.A.); ah.rahmani@qu.edu.sa (A.H.R.); smtrody@qu.edu.sa (S.A.A.); f_alrumaihi@qu.edu.sa (F.A.); 5 Department of Oral Pathology, St. George Hospital Campus, Government Dental College & Hospital, Mumbai 400001, India; anuradhasinghgdc@gmail.com; 6 Department of Computer Science, Maitreyi College, University of Delhi, New Delhi 110021, India; shweta.sank@gmail.com

**Keywords:** module, survival rate, weighted network, key genes, protein–protein interaction

## Abstract

**Simple Summary:**

In this study, four OSCC-specific hub genes were identified using high-throughput RNA-Seq data from TCGA cohort. The significant genes within tumor and normal samples were used for weighted PPI network construction based on survival of patients along with their expression profiles. The analysis revealed the most significant module in the training and test datasets. The genes from this module were used for pathway enrichment analysis followed by hub gene selection. These novel biomarkers might have clinical utility for diagnosis and prognosis prediction in OSCC, providing diagnosis at a very early stage. Moreover, a combination of all these biomarkers might distinguish the OSCC patients with low risk and high risk for cancer progression and recurrence, which will provide useful guidance for personalized and precision therapy. However, the results in the present study were obtained by integrative theoretical analysis, and the findings remain to be confirmed by further experimental validations.

**Abstract:**

Head and neck squamous cell carcinoma (HNSC) is one of the most common malignant tumors worldwide with a high rate of morbidity and mortality, with 90% of predilections occurring for oral squamous cell carcinoma (OSCC). Cancers of the mouth account for 40% of head and neck cancers, including squamous cell carcinomas of the tongue, floor of the mouth, buccal mucosa, lips, hard and soft palate, and gingival. OSCC is the most devastating and commonly occurring oral malignancy, with a mortality rate of 500,000 deaths per year. This has imposed a strong necessity to discover driver genes responsible for its progression and malignancy. In the present study we filtered oral squamous cell carcinoma tissue samples from TCGA-HNSC cohort, which we followed by constructing a weighted PPI network based on the survival of patients and the expression profiles of samples collected from them. We found a total of 46 modules, with 18 modules having more than five edges. The KM and ME analyses revealed a single module (with 12 genes) as significant in the training and test datasets. The genes from this significant module were subjected to pathway enrichment analysis for identification of significant pathways and involved genes. Finally, the overlapping genes between gene sets ranked on the basis of weighted PPI module centralities (i.e., degree and eigenvector), significant pathway genes, and DEGs from a microarray OSCC dataset were considered as OSCC-specific hub genes. These hub genes were clinically validated using the IHC images available from the Human Protein Atlas (HPA) database.

## 1. Introduction

Head and neck squamous cell carcinoma (HNSC) arising from the oral mucosal epithelium, pharynx, and larynx is substantially associated with smoking, tobacco, and human papillomavirus (HPV); hence, it requires multidisciplinary care [1]. HNSC, being one of the most common malignant tumors worldwide with a high rate of morbidity and mortality, has more than 90% of predilections for oral squamous cell carcinoma (OSCC). Cancers of the mouth account for 40% of head and neck cancers, including squamous cell carcinomas of the tongue, lips, buccal mucosa, floor of the mouth, hard and soft palate, and gingival [2,3]. Oral carcinogenesis possesses a series of stages of progression, which simultaneously entail invasion, metastasis, and precancerous lesions. Hence, there is deficiency in the control components of tissues to act in their typical capacities, due to degradation of the cell cycle and the uncontrolled growth of malignant cells. Oral malignant growth is accepted to be a preventable condition because of the chance of early detection and treatment [4]. OSCC, the most devastating and commonly occurring oral malignancy, accounts for 95% of all oral cancer, with a mortality rate of 500,000 deaths per year [5]. Therefore, distinguishable behavioral and molecular etiopathogenesis may challenge accurate prognostication and therapeutics and, therefore, better comprehension of the molecular mechanisms behind the commencement and progression of oral cancer is of extreme significance.

Nowadays, bioinformatics provide a platform for clinical researchers and molecular biologists to switch effortlessly between the clinical practice, laboratory bench, and the use of these sophisticated computational tools, thereby maximizing the advantages brought by computational biology. In last decades, high-throughput (HT) data-based studies such as The Cancer Genome Atlas (TCGA) have enabled the drawing of detailed molecular maps of several cancer diseases, including HNSC, and enhanced discovery of key genes in the pathogenesis of cancer and other diseases, including HNSC [6,7,8]. Similarly, numerous studies have exhibited the function of the RNA sequencing technique in cancers including HNSC, and exposed differential gene expression patterns of prognostic and therapeutic potential [9,10,11]. Detection of altered gene expression patterns in cancer helps in finding key biological pathways, leading to improved insightful understanding of molecular mechanisms of the disease and can be used in precise therapeutic attention in management of the disease [12,13,14]. In this continuation, there is lot of study in the field of HNSC already done and differentially expressed genes (DEGs) have been identified using several techniques. Here, in our work, we identified DEGs with the inclusion of the impression of survival of patients as well as co-expression of genetic profiles with the integration of protein–protein interaction (PPI). Consideration of co-expression is done because, typically, genetic variations in cancer cells which enable altered gene expression patterns can be identified long before the cancer phenotype has established. However, to date, no single gene has shown ample diagnostic utility, and therefore diagnosis and treatment will need to consider the combined influence of many genes [15,16]. There are ten hallmarks of cancer seen during the multistep development of human tumors. These are: self-sustenance in growth signals; resistance to anti-growth signals; eluding apoptosis; unbounded replicative immortality; sustained and uninterrupted angiogenesis; metastasis to distant sites and invasion of local tissues; abnormal metabolic pathways; genome instability; evasion of immune system; and inflammation [17].

Hence, this intensive work has been contributed to illuminate the etiology of OSCC and the important role of a single genetic abnormality in pathogenesis; the molecular mechanisms involved in carcinogenesis and progression required for better understanding of potential diagnostic and therapeutic targets, key functional pathways associated with oncogenesis, and the perturbations of interactions in the complex network still remain complicated. Thus, we conducted this study for the identification of specific biomarkers of OSCC, by creating weighted PPI of DEGs followed by detection of modules in an updated weighted PPI network algorithm where weight indicated the differential co-efficient of co-expression on survival (shorter vs. longer), which may contribute to developing effective diagnostic, therapeutic, and prognostic strategies. Furthermore, we identified hub genes from significant modules based on topological characteristics of the weighted PPI network, gene enrichment, and pathway analyses. Finally, four significant signature genes (ISG15, OASL, IFI6, and RSAD2) related to patient survival were identified based on gene expression profiling data extracted from TCGA database. Out of the four, ISG15 had the highest weighted degree and eigenvector measure which is associated to OSCC and highly significant. These findings emphasized that untangling the network-based survival-associated module may contribute to biomarker-guided preclinical and modality of clinical therapeutic development.

## 2. Materials and Methods

### 2.1. TCGA RNA-Seq Data Extraction and Differential Expression Analysis

Messenger RNA (mRNA) HTSeq raw count data (based on IlluminaHiSeq platform) of TCGA-HNSC cohort was retrieved from UCSC Xena browser (https://xenabrowser.net/, accessed on 1 June 2021) [18]. These samples were then verified with respect to mRNA-Seq HNSC samples present in TCGA GDC data portal (https://portal.gdc.cancer.gov/, accessed on 1 June 2021) and samples pertaining to OSCC-specific areas (i.e., floor of mouth, base of tongue, gum, palate, other and unspecified parts of mouth, and other and unspecified parts of tongue) were retained. The clinical survival data of these OSCC samples were retrieved from Xena and only overlapping samples from both the count and survival datasets were retained. The raw counts corresponding to solely primary solid tumor and normal solid tissue samples were back-log-transformed to obtain raw integer counts. DESeq2 R package [19] was used for obtaining normalized and $\log_{2}$-transformed expression values through variance stabilizing transformation (VST) of mRNA integer count data. The ARSyNseq function in the NOISeq R package [20] was used for batch effect correction in normalized expression values with unknown batch setting. The biomaRt package [21,22] was used for mapping the Ensembl IDs to their corresponding HGNC symbol(s). Expression values of a gene mapping to multiple Ensembl IDs were taken as an average to avoid redundancy [13,14,23]. DEGs were detected using Limma R package [24] corresponding to a threshold of $\left|\log_{2}\left(\mathrm{fold}\;\mathrm{change}\right)\right|>2$ with Benjamini–Hochberg (BH)-adjusted *p*-value < 0.05.

### 2.2. PPI Network and Its Weighted Form

PPI network of the OSCC-associated DEGs was constructed using Search Tool for the Retrieval of Interacting Genes (STRING, https://string-db.org/, accessed on 1 June 2021) v11.0 database [25], considering interactions with a confidence score > 0.9. The PPI network consists of nodes of proteins, and their predicted interacting protein edges are without weight. This PPI is converted into a weighted PPI with the inclusion of differential coefficients in co-expressions of pairs of proteins in longer and shorter survival patients. Each weight for a pair of interacting proteins denoted a co-expression coefficient of differentiation between longer and shorter survival patients in the training dataset. The data of all patients are randomly divided in two parts; one is known as the training dataset (60% of the total sample), and the other is known as the test dataset (40% of the total sample). The weighted PPI network for both datasets separately were constructed utilizing the methodology adopted from [26], in which each edge was allocated a weight, $D_{xy}$, on the basis of its degree of differential co-expression of a pair genes (genes *x* and *y*) between longer and shorter survival samples, as follows:

(1) $$D_{xy}=\frac{\left|Z_{r}{}_{L}-Z_{r}{}_{S}\right|}{\sqrt{\frac{1}{n_{L}-3}+\frac{1}{n_{S}-3}}}$$

where $Z_{r}{}_{L}$ and $Z_{r}{}_{S}$ are Fisher’s *Z* transformation of Pearson’s correlation coefficients $r_{L}$(correlation coefficients between *x* and *y* genes in longer survival data) and $r_{S}$ (correlation coefficients between x and y genes in shorter survival data).

Fisher’s *Z* transformation has been done using the following formula:

(2) $$Z_{r}=\frac{1}{2}\ln\left(\frac{1+r}{1-r}\right)$$

Consequently, lower value of $D_{xy}$ concludes similar correlation in both shorter and longer survival samples, which is not significant; in contrast, higher value of $D_{xy}$ is significant. Thus, weight on each pair is proportional to the correlation with survival of patients, offering a weighted PPI network for further analysis.

### 2.3. Module Detection in Weighted PPI Network

The determination of the pool of proteins with high $D_{xy}$ is another challenge which is also known as module detection in a weighted PPI network. Therefore, module was detected using a neighborhood proximity-based algorithm for overlapping community structure detection in weighted networks [27]. This is a recent improved algorithm for the weighted network, providing multiple membership of nodes more realistic than the classical module detection algorithms such as [28,29,30], etc. This algorithm will provide communities based on the weight of each pair. This weight is already inbuilt with the information associated with the survival of patients as well as the correlation of gene expression between pairs of proteins.

### 2.4. Survival Analysis of Modules

Furthermore, each module needs exploratory survival analysis in the training and test datasets to determine the highly significant module based on the survival data. For this, we did the analysis in two phases. In phase one, we did principal component analysis (PCA) of the gene expression data of the specific set of genes from a module. This analysis provided the most representative gene expression in a module [16], known as module eigengene (ME). The module eigengene (ME) was calculated using singular value decomposition (SVD), which is explained in [31]. Thus, the samples were split into two groupings based on the median value of ME. In phase two, survival curves were estimated by the Kaplan–Meier (KM) method for each group of samples and compared with the log-rank test. Consequences of both phases observed the significance of survival-based modules in the training and test datasets separately.

### 2.5. OSCC-Specific Hub Gene(s) Detection

OSCC-associated survival-based significant module genes were further subjected to pathway enrichment analysis using the Enrichr database (https://maayanlab.cloud/Enrichr/, accessed on 1 June 2021) [32]. The Reactome library available within the Enrichr database was used and the top 10 significant pathways corresponding to *p*-value < 0.05 were selected. Afterwards, National Center for Biotechnology Information (NCBI)-Gene Expression Omnibus (GEO) (https://www.ncbi.nlm.nih.gov/geo/, accessed on 1 June 2021) [33] was queried by using “OSCC” and “Oral Squamous Cell Carcinoma” as suitable keywords to extract OSCC-associated mRNA expression profiles. The search results were further trimmed down by applying inclusion criteria: (1) the dataset should be “expression profiling by array” type and its samples should belong to “Homo Sapiens”; (2) the dataset must have processed and raw microarray data; (3) the dataset must have paired tumor and normal samples; and (4) the dataset must have greater than 20 samples. The GEO2R (https://www.ncbi.nlm.nih.gov/geo/geo2r/, accessed on 1 June 2021) web-based tool was used for detecting DEGs between the paired sample groups. The genes were regarded as differentially expressed corresponding to BH-*p*-value < 0.01 and $\left|\log_{2}\left(\mathrm{fold}\;\mathrm{change}\right)>1.5\right|$. CytoNCA (plugin available within Cytoscape) [34] was used to analyze the centrality measures of the chosen weighted PPI module. Gene sets ranked on the basis of weighted PPI module centralities, the top 10 significant pathway genes, and DEGs from the OSCC microarray dataset were used to identify the OSCC-specific hub genes. The protein expression pattern of these genes in tumor and normal tissues was validated using the Human Protein Atlas (HPA) database (https://www.proteinatlas.org/, accessed on 1 June 2021) [35,36,37,38,39,40].

## 3. Results

### 3.1. TCGA RNA-Seq Processing and Differential Expression Analysis

**Figure 1:**
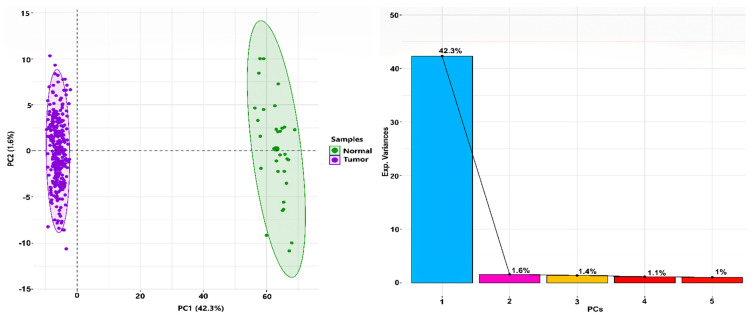
PCA plot in the left panel representing the variation in the expression data between normal and tumor samples. Each point in the plot shows the overall expression value of 916 DEGs. The color of each point represents the disease status: green for normal and magenta for tumor. The percentages of total variation across the first two principal components (PCs) are shown on the *x* and *y* axes, respectively. It can be observed that both the normal and tumor samples are clustered independently and distinctly. Scree plot in the right panel showing percentage of explained variances (on *y*-axis) captured by their corresponding PCs (on *x*-axis).

**Figure 2:**
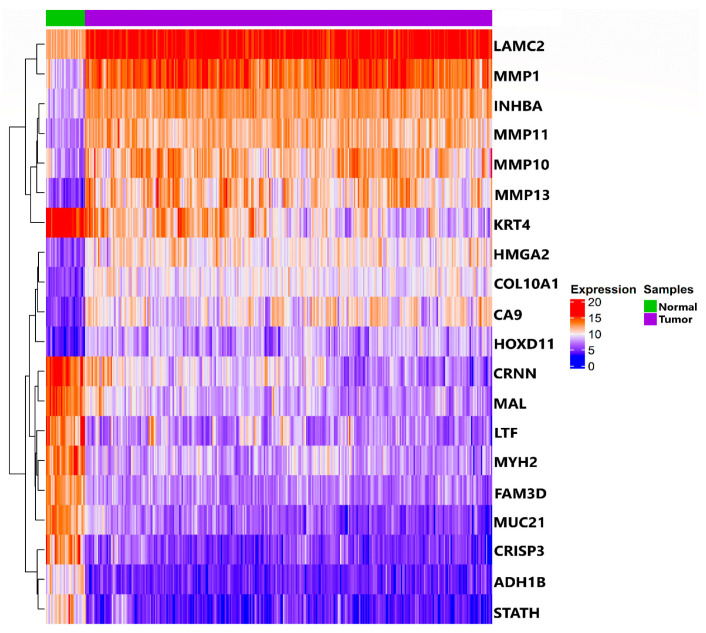
Heatmap of the top 10 up and downregulated OSCC-associated DEGs. Hierarchical clustering using Pearson distance was applied for rows with its corresponding cluster dendrogram being displayed along the left side of the plot, respectively. The column annotation bar depicting status of samples (i.e., green for normal and magenta for tumor) was shown at the top of the heatmap.

OSCC-specific count data (filtered from TCGA-HNSC cohort) had a total of 352 samples (i.e., 321 tumor and 31 healthy normal samples). After performing normalization, $\log_{2}$ transformation, and batch correction, a total of 51,841 Ensembl IDs mapped to their corresponding HGNC symbol(s). After averaging expression values of a gene mapping to multiple Ensembl IDs, we were left with a total of 50,683 unique genes and their respective expression values across the samples. We obtained 916 DEGs in accordance with the aforementioned threshold, i.e., $\left|\log_{2}\left(\mathrm{fold}\;\mathrm{change}\right)\right|>2$ and BH-*p*-value < 0.05 using limma. A total of 266 and 650 DEGs were filtered as up and downregulated, respectively. Figure 1 shows a 2D PCA plot (on left panel) exhibiting the clusters and variations in DEGs between tumor and normal samples across the first two principal components (PCs), whereas the scree plot (on right panel) represents the percentage of explained variances accounted for by the first five PCs. Figure 2 shows an expression heatmap plot of the top 10 up and downregulated DEGs across (primary solid) tumor and (solid tissue) normal samples, respectively.

### 3.2. Construction of Weighted PPI Network and Module(s) Detection

**Figure 3:**
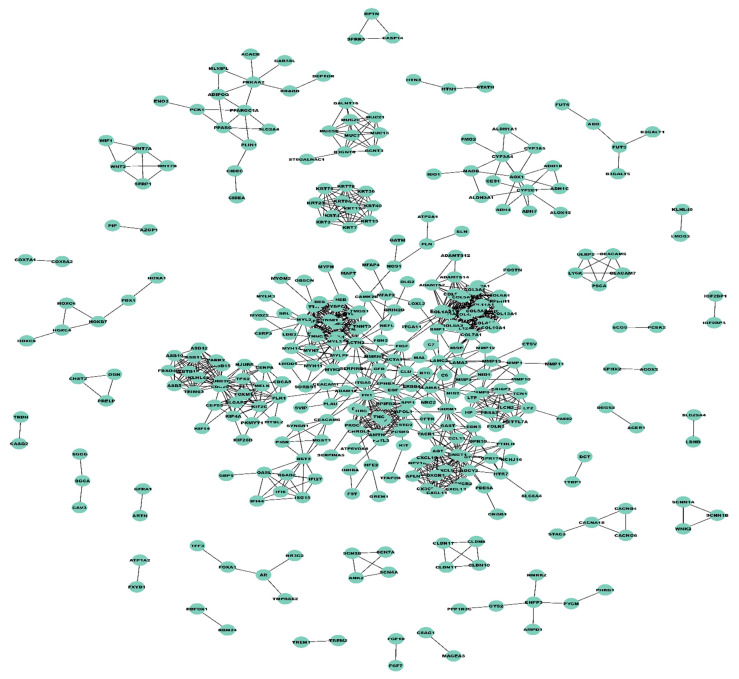
Weighted PPI network comprising 346 nodes and 1207 edges, visualized using Cytoscape.

Unweighted PPI network constructed utilizing STRING involved a total of 346 nodes and 1207 edges corresponding to a confidence score > 0.9. The unweighted PPI was converted into a weighted PPI network (Figure 3) where weights were created using mRNA expression profiles from 218 training samples out of 352 total samples. These 218 samples were considered for the training dataset and remained for the test dataset. A neighborhood proximity-based algorithm was used to determine the number of modules in the weighted PPI network. This algorithm yielded a total of 46 modules in the constructed weighted PPI network, out of which eighteen had more than five nodes. These 18 modules were used for further analysis. The list of genes in each module can be seen in Supplementary Table S1 (here each row represents a module).

### 3.3. Module Survival Analysis

**Figure 4:**
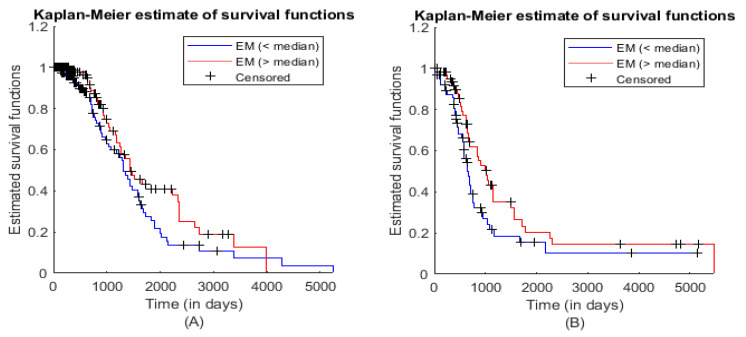
KM plots with log-rank test for module number 5 in (**A**) training and (**B**) test datasets.

**Figure 5:**
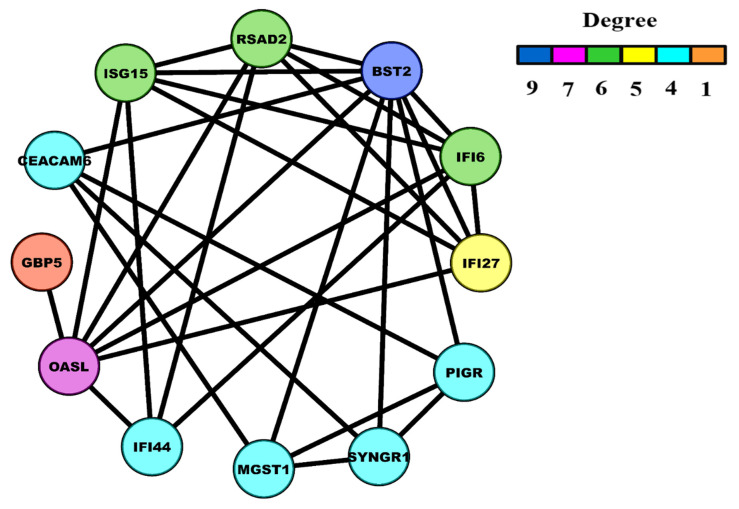
Weighted PPI module with 12 nodes and 30 edges. The color of nodes varies in accordance with their degrees.

We then analyzed all 18 significant modules. ME was calculated for each module as explained in the Materials and Methods section, and significant module(s) in the training and test datasets were identified. Module numbers 4, 5, 10, 18, 24, 37, and 41 were significant (*p*-value computed using log-rank test) in the training dataset, whereas module numbers 5 and 36 were significant in the test dataset. Module number 5 was common in both the training and test datasets. This significant module had 12 genes (list of genes can be seen in 5th row of Supplementary Table S1). KM plot and log-rank test of module number 5 for the training and test datasets can be seen in Figure 4A,B and corresponding statistical values can be seen in Table 1 and Table 2, respectively. Weighted PPI network module comprising 12 nodes and 30 interaction edges can be seen in Figure 5.

### 3.4. HNSC-Specific Hub Gene(s) Detection

**Figure 6:**
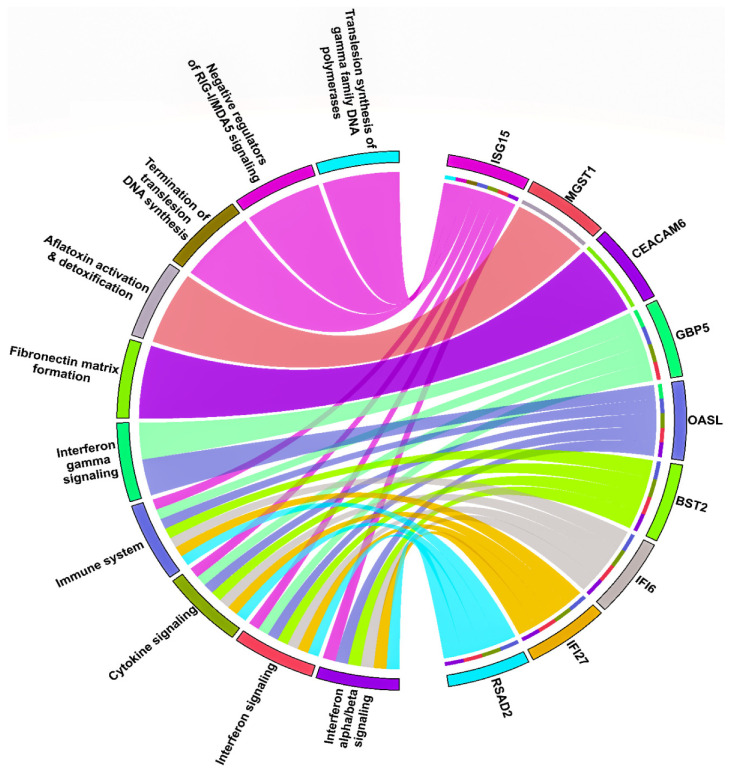
Chord plot showing the association of the top 10 significant pathways and nine DEGs involved in them. The outer circle indicates the top 10 pathways (on the left semicircle) and nine DEGs (on the right semicircle). Each DEG has a unique color band, and the undirected colored edge inside the circle represents the relationship of a particular DEG with the respective connected pathway(s).

**Figure 7:**
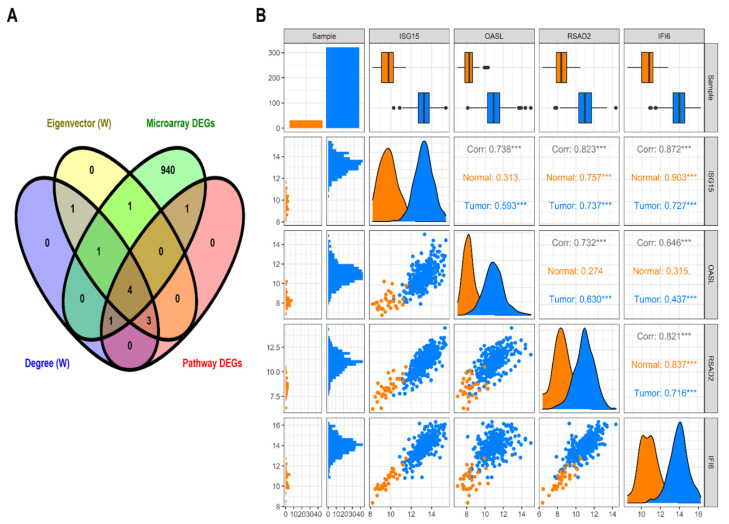
OSCC-specific hub genes and association between them. (**A**) Venn plot showing four overlapping OSCC-specific hub genes between the top 10 ranked genes (based on weighted degree and weighted eigenvector), top 10 significant pathway genes (nine), and OSCC microarray DEGs (948). Yellow, violet, red, and green colored areas in the Venn plot represent gene sets corresponding to weighted eigenvector, weighted degree, pathway DEGs, and OSCC microarray DEGs. (**B**) Pairwise scatter plot showing the associations amongst these four upregulated OSCC-specific hub genes. The upper triangular section represents the Spearman correlation coefficients between these hub genes along with expression boxplots for each gene. The lower triangular section represents the scatterplot and histogram distribution between these genes. The diagonal consists of kernel densities for each gene. The asterisk (***) sign indicates the moderate and higher correlation values.

**Figure 8:**
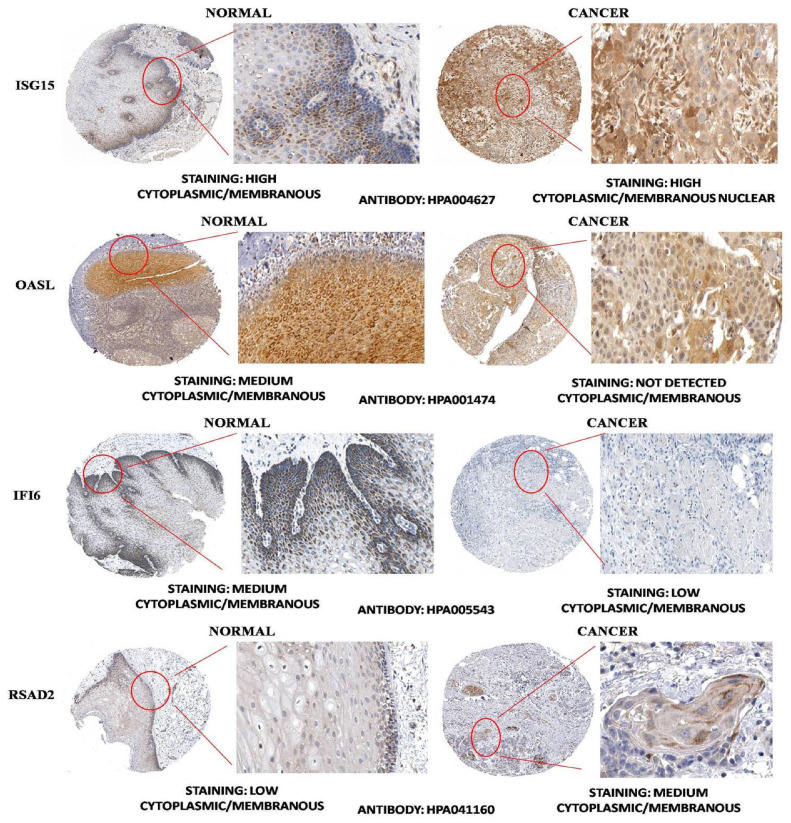
Representative immunohistochemistry images of ISG15, OASL, IFI6, and RSAD2 in normal head and neck tissues and head and neck squamous cell carcinoma (HNSC) tissues (Human Protein Atlas database).

A total of nine module genes out of twelve were involved in the top 10 significant pathways, and their interactions can be seen via a chord plot in Figure 6. Based on the specified searching and inclusion criteria, we chose the OSCC mRNA expression profile possessing accession number GSE37991. It comprised 80 paired samples of OSCC tumor and adjacent non-tumor tissues. A total of 948 genes were differentially expressed corresponding to BH-*p*-value < 0.01 and $\left|\log_{2}\left(\mathrm{fold}\;\mathrm{change}\right)\right|>1.5$. With the help of CytoNCA we ranked the top 10 weighted PPI module DEGs based on their degree and eigenvector centralities. The Venn plot as shown in Figure 7A exhibits the sets comprising the top 10 ranked genes (based on weighted degree and eigenvector), significant pathway DEGs (i.e., nine DEGs), and DEGs from the OSCC microarray dataset (validation set). The overlapping four DEGs (i.e., ISG15, OASL, IFI6, and RSAD2) were termed as the OSCC-specific hub genes. Out of all these, ISG15 had the highest weighted degree and eigenvector measure. A pairwise scatter plot matrix exhibiting the association among these four upregulated hub genes is shown in Figure 7B. Within the plot, the highest correlation of 0.872 was observed between ISG15 and IFI6, followed by a correlation of 0.823 between ISG15 and RSAD2, respectively. We then tried to analyze the protein expression patterns of these four DEGs (i.e., ISG15, OASL, IFI6, and RSAD2) in normal head and neck tissues and HNSC tissues by using the HPA database (Figure 8). High immunoexpressions of ISG15 were observed in both normal and carcinoma tissues. In addition, medium protein expressions of OASL and IFI6 were seen in normal tissues, however no immunoexpressions of OASL and low expression of IFI6 were observed in HNSC tissues. Additionally, low and medium protein expressions of RSAD2 were seen in normal and tumor tissues, respectively.

## 4. Discussion

In the present study, 352 samples were included. Based on gene expression datasets, a total of 916 DEGs were obtained, consisting of 266 upregulated genes and 650 downregulated genes. Our results revealed that nine DEGs were associated with the top 10 significant pathways. The most upregulated four DEGs i.e., interferon-stimulated gene 15 (ISG15), oligoadenylate synthetase-like gene (OASL), interferon alpha-inducible gene 6 (IFI6), and radical S-adenosyl methionine domain containing 2 gene (RSAD2) were termed as the OSCC-specific hub genes, of which ISG15 had the highest weighted degree and eigenvector measure. These genes are mostly anti-viral immunomodulators and are generally linked with immune and cytokine interferon-mediated signaling pathways. Although mechanistic insights into the liaison between these hub genes and several types of diseases have been extensively investigated, their diagnostic, therapeutic and prognostic roles in OSCC still remain largely unexplored. Thus, the application of multiple bioinformatic strategies and publicly available gene expression profiles may provide practicable and definitive approaches for the better understanding of these tumor biomarkers in OSCC, which may further help further enlighten tumor molecular genetics.

ISG15, a ubiquitin-like protein, is immunomodulatory in function, whereby it stimulates IFN-gamma production and augments the proliferation of natural killer (NK) cells. The ISGylation process regulates diverse cellular pathways such as cytoskeleton organization, RNA splicing, chromatin remodeling/polymerase II transcription and regulation, stress responses, translation, cell proliferation, signal transduction, and apoptosis [41]. Several studies have revealed enhanced expression of ISG15 and deregulation of enzymes that catalyze ISGylation and de-ISGylation in many types of cancers, involving bladder cancer, breast cancer, prostate cancer, hepatocellular carcinoma, colorectal cancer, nasopharyngeal carcinoma, gastric cancer, oesophageal squamous cell carcinoma [42], and OSCC [43,44]. Therefore, deregulation in the expression of ISG15 may be expected to have pro-tumor functions, and thus an increased ISG15 level may promote carcinogenesis, giving the possibility of its use as a high-confidence diagnostic, therapeutic, and immunostimulant tumor biomarker [41,42].

OASL, one of oligoadenylate synthetase’s (OAS’s) family members, belongs to a template-independent nucleotidyltransferase family and has interferon-induced antiviral activity, and can therefore aid in cancer immunotherapy. OASL lacks the ability to synthesize 2′-5′-oligoadenylate, which is different from other OAS family members [45]. The OASL gene has been found to be associated with the regulation of lung cancer cell sensitivity to acRoots, via the PI3K signal pathway [46]. OASL has also been associated with proliferation of cancer such as that of the gastric and breast type, and therefore can play a role in prognostication with potential mechanistic value in breast cancer [47]. Lohavanichbutr P et al., using L1/L2-penalized Cox regression models in HPV-negative OSCC, identified six genes, one of which was OASL, and reported that all these genes play a role in cell invasion and motility, cell-to-cell signaling, signal transduction, and proliferation, processes essential to metastasis and cancer progression [48].

IFI6, also known as GIP3, which belongs to the ISG12 gene family, has been reported as a mitochondrial and apoptotic protein in myeloma, gastric, and breast cancer, and therefore IFI6-induced mitochondrial redox deregulation bestows metastatic potentials in these cancers [49]. It has been found that IFI6 can be one of the potential biomarkers of OSCC [50,51]. A high level of expression of IFI6 has been seen in colorectal cancer, gastric cancer, breast cancer, myeloma, and tongue squamous cell carcinoma; additionally, expression is extremely high in multi-drug resistant cancer cells, suggesting that a close correlation between IFI6 levels and resistance to apoptosis is present [52,53,54].

RSAD2 is an endoplasmic reticulum (ER)–associated, interferon-inducible anti-viral protein [55]. It has been identified as a potential biomarker for prognostication in various cancers including OSCC [56,57].

## 5. Conclusions

In conclusion, the present study was done to identify DEGs in OSCC, and to explore the underlying mechanisms of tumorigenesis by using integrated bioinformatics analysis. We identified four hub genes (ISG15, OASL, IFI6, and RSAD2) with the highest expression of ISG15, which may fill in as original and novel biomarkers, and remedial focuses for the exact conclusion and treatment of OSCC later on. When contrasted with single-dataset examinations, our investigation gives more solid and exact outcomes by utilizing a few datasets. However, the data in the present study were obtained by analysis of the theoretical approach of bioinformatics, and the findings remain to be confirmed by further investigations. Therefore, further experimental validation is warranted to elucidate and ascertain the clinical value of the identified genes as biomarkers in addition to the underlying mechanisms. Furthermore, other non-genomic regulatory factors, epigenetic alterations, and re-arrangement mechanisms may be involved to increase gene expression. These outcomes will surely add to the current information on oral carcinogenesis and may be helpful for future application in the visualization and treatment of OSCC.

## Figures and Tables

**Table 1:** Statistical values of log-rank test for the training dataset corresponding to Figure 4A.

Statistical Characteristics	Samples for ME (<Median)	Samples for ME (>Median)
Hazard Rate	0.0047	0.0040
95% Confidence Interval	0.9615 (Lower limit)	2.1424 (Upper limit)
Hazard Ratio	1.4353	
z-value	1.66584	
*p*-value (two-tailed test)	0.09575	

**Table 2:** Statistical values of log-rank test for the test dataset corresponding to Figure 4B.

Statistical Characteristics	Samples for ME (<Median)	Samples for ME (>Median)
Hazard Rate	0.0072	0.0065
95% Confidence Interval	1.0084 (Lower limit)	2.4600 (Upper limit)
Hazard Ratio	1.5750	
z-value	1.88315	
*p*-value (two-tailed test)	0.05968	

## Data Availability

The raw HTSeq count data of TCGA-HNSC cohort used in our study was downloaded from UCSC Xena browser (https://xenabrowser.net/datapages/?cohort=GDC%20TCGA%20Head%20and%20Neck%20Cancer%20(HNSC)&removeHub=https%3A%2F%2Fxena.treehouse.gi.ucsc.edu%3A443, accessed on 1 June 2021). The OSCC mRNA expression dataset used in our study was downloaded from the Gene Expression Omnibus (GEO) under accession number GSE37991 (https://www.ncbi.nlm.nih.gov/geo/query/acc.cgi?acc=GSE37991, accessed on 1 June 2021).

## Supplementary Materials

The following are available online at https://www.mdpi.com/article/10.3390/biology10080760/s1, Table S1: Number of modules in weighted PPI network and their number of genes.
